# Loss of DLG5 promotes breast cancer malignancy by inhibiting the Hippo signaling pathway

**DOI:** 10.1038/srep42125

**Published:** 2017-02-07

**Authors:** Jie Liu, Juan Li, Pingping Li, Yaochun Wang, Zheyong Liang, Yina Jiang, Jing Li, Chen Feng, Ruiqi Wang, He Chen, Can Zhou, Jianmin Zhang, Jin Yang, Peijun Liu

**Affiliations:** 1 Center for Translational Medicine, the First Affiliated Hospital of Xi’an Jiaotong University, Xi’an, Shaanxi, China; 2 Key Laboratory for Tumor Precision Medicine of Shaanxi Provincer, the First Affiliated Hospital of Xi’an Jiaotong University, Xi’an, Shaanxi, China; 3 Department of Pathology, the First Affiliated Hospital of Xi’an Jiaotong University, Xi’an, Shaanxi, China; 4 Department of Oncology, the Shaanxi Provincial Corps’ Hospital, Xi’an, Shaanxi, China; 5 Department of Breast Surgery, The First Affiliated Hospital of Xi’an Jiaotong University, Xi’an, Shaanxi 710061, China; 6 Department of Cancer Genetics, Roswell Park Cancer Institute, Buffalo, New York, USA; 7 Department of Medical Oncology, the First Affiliated Hospital of Xi’an Jiaotong University, Xi’an, Shaanxi, China

## Abstract

Discs Large Homolog 5 (DLG5) plays an important role in the maintenance of epithelial cell polarity. Recent research showed that DLG5 is decreased in Yes-associated protein (YAP)-overexpressing cells. However, the exact relationship between DLG5 and YAP is not clear. In this study, we showed that loss of DLG5 promoted breast cancer cell proliferation by inhibiting the Hippo signaling pathway and increasing nuclear YAP expression. Furthermore, depletion of DLG5 induced epithelial-mesenchymal transition (EMT) and disrupted epithelial cell polarity, which was associated with altered expression of Scribble, ZO1, E-cadherin and N-cadherin and their mislocalization. Interestingly, we first reported that loss of DLG5 inhibited the interaction of Mst1 and Lats1 with Scribble, which was crucial for YAP activation and the transcription of TEA domain (TEAD) family members. In summary, loss of DLG5 expression promoted breast cancer malignancy by inactivating the Hippo signaling pathway and increasing nuclear YAP.

The Hippo signaling pathway is an evolutionarily conserved kinase cascade involved in organ size control, tissue homeostasis and cancer^1^,^2^. Yes-associated protein (YAP) is a major effector of Hippo signaling; it interacts with the DNA-binding transcription factor TEAD and is closely related to cell proliferation, survival, migration, and invasion^3^,^4^. Studies have shown that inactivation of the Hippo signaling pathway and YAP nuclear localization are closely associated with multiple epithelial malignancies, such as breast cancer^5^,^6^. However, genomic analyses of common epithelial cancers have provided no evidence of an increased number of mutations in known components of the Hippo signaling pathway^3^,^7^. Therefore, alterations in the Hippo signaling pathway in human cancer might be caused by mutations in genes associated with this pathway but not by mutations in its own components.

Discs large homolog 5 (DLG5) is a primary member of the membrane-associated guanylate kinase (MAGUK) family, and its expression is decreased in YAP-overexpressing cells^8^. DLG5 also participates in the maintenance of epithelial polarity by interacting with β-catenin, the vinexin-vinculin complex and cadherin and by promoting the delivery of N-cadherin to the plasma membrane^9^,^10^,^11^. According to several publicly available data sets, DLG5 is also associated with cancer development. DLG5 is highly expressed in normal tissues, but its expression is decreased or lost in cancer cell lines. Down-regulation of DLG5 is highly correlated with clinical tumor stage. In breast cancer, knockdown of DLG5 induces cell migration, and overexpression of DLG5 inhibits cell migration^8^. However, the detailed relationship between DLG5 and YAP in human carcinogenesis has not been investigated.

We intend to verify the connection of DLG5 and YAP in breast cancer development. In our study, we first found that loss of DLG5 inhibited the Hippo pathway by decreasing the phosphorylation of MST1/2, LATS1, and MOB1 and by increasing YAP nuclear localization; loss of DLG5 also promoted the transcription of TEAD-target genes. Interestingly, loss of DLG5 expression promoted cell proliferation, which was associated with the down-regulation and mislocalization of Scribble and decreased interaction of MST1 and Lats1 with Scribble. Our study revealed that knockdown of endogenous DLG5 inhibited the Hippo signaling pathway, induced EMT, disrupted epithelial cell polarity, and enhanced cell migration and invasiveness, thereby promoting breast cancer malignancy.

## Materials and Methods

### Patients and tissue specimens

This work was conducted in accordance with the Code of Ethics of the World Medical Association. With the approval of the Ethics Review Committee of the First Affiliated Hospital of Xi’an Jiaotong University College of Medicine, a total of 75 breast cancer tissue specimens and adjacent normal control tissues were obtained from the First Affiliated Hospital of Xi’an Jiaotong University College of Medicine (sTable 1).

### Antibodies and Reagents

Antibodies used in this study included anti-DLG5 (Sigma), anti-N-cadherin (BD transduction), anti-E-cadherin (BD Biosciences), anti-vimentin (Sigma), anti-ZO1 (Sigma) and Hippo Signaling Antibody Sampler Kit (CST).

### Cell culture

MCF10A cells were cultured as previously described^12^. MCF7, T47D and MDA-MB-231 cells were grown according to the recommendations of American Type Culture Collection (ATCC).

### Three-dimensional (3D) morphogenesis assay

MCF10A cells were cultured in Growth Factor-Reduced BD Matrigel^TM^ (BD Biosciences) in a 4-well chamber slide (Corning) as previously described^13^,^14^.

### Lentivirus packaging and infection

The lentivirus system included the pLKO.1, VSVG and pCMVΔR8.9 plasmids for expressing short hairpin RNA (shRNA); these plasmids were transfected into HEK-293T cells. Then, virus-containing culture medium was harvested. The target cells were incubated with harvested medium containing polybrene (Sigma). Then, colonies were isolated, expanded, and maintained.

### Immunofluorescence microscopy

The cells were fixed, permeabilized, blocked and incubated with primary antibodies. The cells were stained with Alexa Fluor-labeled secondary antibody (Invitrogen). Fluorescence images were obtained using a confocal laser scanning microscope (Leica TCS SP5).

### Immunohistochemistry (IHC)

IHC images were obtained using a microscope slide scanner (Leica MP, SCN400). DLG5 expression levels in tissue were assessed based on positive staining and rated as 0 (negative), 1 (weakly positive), 2 (moderately positive), or 3 (strongly positive).

### Western blotting

Cells were harvested in RIPA buffer containing protease inhibitors. Cell lysates were loaded onto gels, and proteins were separated and then transferred onto PVDF membranes (Millipore). Bands were developed with western blotting luminol reagent (Millipore).

### Flow cytometry analysis

Flow cytometry analysis was used to examine cell cycle characteristics. Cells were fixed and stained with propidium iodide (PI). Cell cycle analysis was performed with a BD FACSCanto II flow cytometer at 488 nm, and data were analyzed using ModFit LT (BD Biosciences).

### BrdU incorporation assay

The assay was performed as previously described and analyzed by flow cytometry^15^.

### Luciferase assay

Luciferase and Renilla reporters were co-transfected into cells, which were analyzed with the Dual-Luciferase^®^ Reporter Assay and Dual-Luciferase^®^ Reporter 1000 Assay Systems (Promega).

### Animals and xenotransplantation

All animal experimental protocols were approved by the Institutional Animal Care and Use Committee of the First Affiliated Hospital of Xi’an Jiaotong University. In addition, the methods were in accordance with the approved guidelines. A total of 5 × 10^6^ resuspended cells were injected subcutaneously into the fat pad of six-week-old female nude mice (Centre of Laboratory Animals, The Medical College of Xi’an Jiaotong University, Xi’an, China). Tumor volume was calculated using the following formula: (long axis × short axis^2^)/2.

### Statistical analysis

Each experiment was repeated at least three independent times. The results are presented as the mean ± SD or SEM as indicated. Statistical analysis was conducted using GraphPad Prism 5, and the results are presented as follows: *p < 0.05, **p < 0.01 or ***p < 0.001.

## Results

### Distribution of DLG5 expression in breast epithelial cells, breast cancer cells, breast cancer tissues and para-cancerous tissues

**Figure 1:**
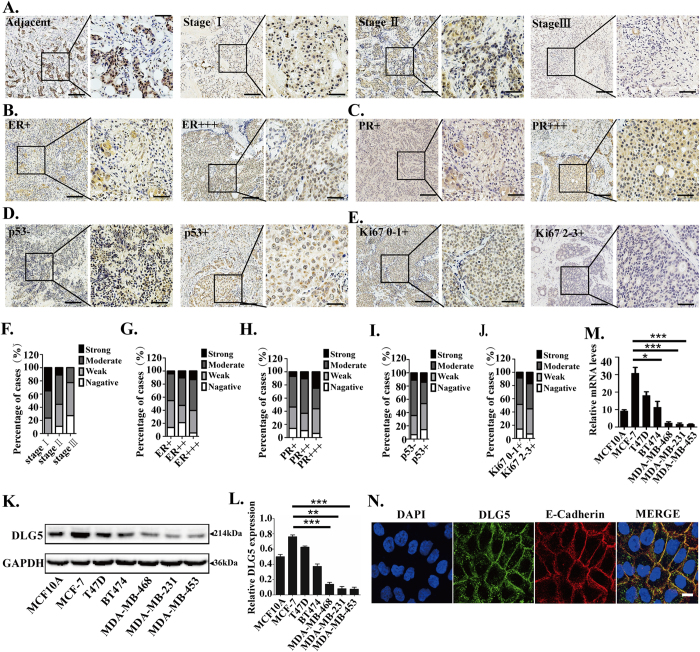
DLG5 expression in tissues and cell lines. Tissues were stained with a DLG5 antibody. Low-power scale bar, 150 μm; high-power scale bar, 50 μm. (**A**) From left to right: normal breast epithelial tissues, stage I breast cancer tissues, stage II breast cancer tissues, and stage III breast cancer tissues. (**B**) Immunostaining of DLG5 in ER+++ and ER+ breast cancer tissues. (**C**) Immunostaining of DLG5 in PR+++ and PR+ breast cancer tissues. (**D**) Immunostaining of DLG5 in Ki67−/+ and Ki67+ breast cancer tissues. (**E**) Immunostaining of DLG5 in p53+ and p53−/+ breast cancer tissues. (**F**) Distribution of specimens at different stages (stage I/II/III) according to DLG5 expression levels. (**G,H,I,J**) Distribution of breast cancer specimens according to DLG5 expression levels and ER/PR, Ki67 and p53 status. (**K**) Western blot analysis of DLG5 expression in different breast cancer cell lines. (**L**) Western blot results quantified using scanning densitometry are presented as a ratio relative to GAPDH. (**M**) DLG5 mRNA levels in different breast cancer cell lines were analyzed by real-time PCR. N. DLG5 and E-cadherin localization in MCF10A cells was analyzed by immunofluorescence staining (scale bar, 25 μm).

To identify the relationship between DLG5 and breast cancer, we examined the expression of DLG5 in breast cancer tissues. DLG5 showed stronger staining in normal mammary tissues than in breast cancer tissues. Low-grade cancer tissues had higher expression of DLG5 than high-grade cancer tissues (Fig. 1A,F). Nearly 76% of stage I breast cancer, 55% of stage II breast cancer, and 23% of stage III breast cancer samples showed moderate or strong DLG5 staining. Of the stage III breast cancer tissues, 50% were weakly positive, and 27% were negative (sTable 2). To summarize, stage I and stage II breast cancer tissues demonstrated reduced DLG5 staining, while stage III tissues showed loss of or reduced DLG5 staining.

Estrogen receptor (ER) and progesterone receptor (PR) status is a biomarker for breast cancer therapy and prognosis^16^,^17^. DLG5 expression showed a significant positive correlation with ER and PR status in breast cancer. Nearly 60% of ER+++ tissues showed strong or moderate DLG5 staining, while over 50% of ER−/+ tissues showed weak or negative DLG5 staining (Fig. 1B,G; sTable 2). Similarly, PR-positive tissues showed stronger DLG5 staining than PR-negative tissues. Strong DLG5 staining was present in more than 25% of PR +++ tissues but in only 7% of PR−/+ tissues. Nearly 50% of PR−/+ tissues displayed weak or negative staining for DLG5 (Fig. 1C,H; sTable 2). The results suggest that DLG5 might be associated with breast cancer therapy and prognosis.

p53 is a tumor suppressor that is important in cell cycle arrest^18^,^19^. Ki67 is a well-known diagnostic marker for various cancers; its expression is positively associated with cell growth and reflects cell proliferation^20^,^21^. We observed stronger DLG5 staining in p53+ tissues than in p53−/+ tissues (Fig. 1D,I; sTable 2). Meanwhile, DLG5 staining was weak or negative in Ki67+ tissues but moderate or strong in Ki67−/+ tissues (Fig. 1E,J; sTable 2). These results suggest that loss of DLG5 expression might be associated with breast cancer cell proliferation.

Breast cancer cell lines showed similar results. A non-transformed breast epithelial cell line (MCF10A) and low-grade breast cancer cell lines (MCF7, T47D and BT474) showed higher DLG5 protein and mRNA expression levels than high-grade breast cancer cell lines (MDA-MB-468, MDA-MB-231 and MDA-MB-453). DLG5 expression was higher in breast cancer cell lines of the luminal subtype than in those of the basal-like subtype (Fig. 1K,L,M). Additionally, DLG5 and E-cadherin co-localized at MCF10A cell-cell junctions (Fig. 1N), which further verified the important role of DLG5 in the maintenance of epithelial cell polarity. These results suggest that DLG5 might participate in breast cancer development.

### Loss of DLG5 promotes cell proliferation

**Figure 2:**
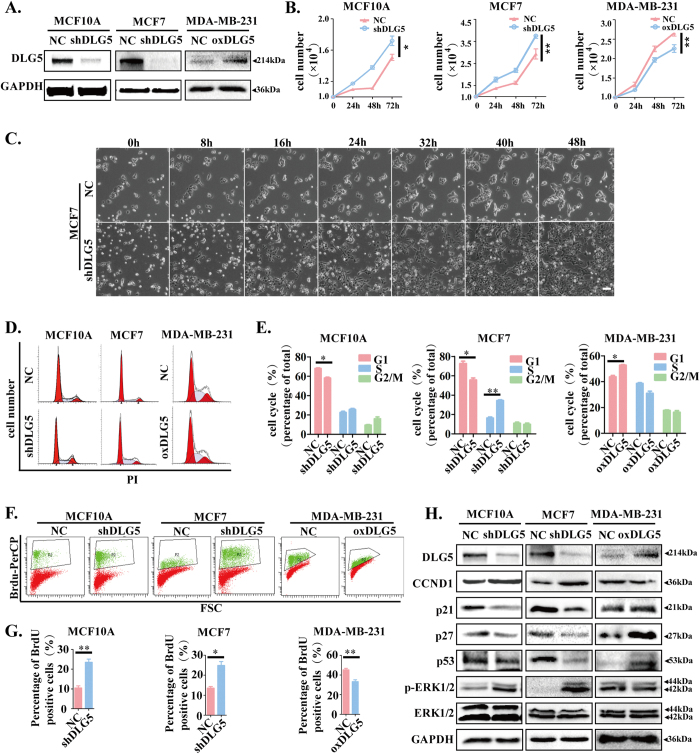
Knockdown of DLG5 promotes cell proliferation. (**A**) Immunoblot analysis of DLG5 expression in NC, MCF10A-shDLG5, MCF7-shDLG5 and MDA-MB-231-oxDLG5 cells. (**B**) The cells were counted at 24, 48 and 72 hrs. (**C**) NC and MCF7-shDLG5 cells were photographed every 8 hrs using a Live Cell Imaging System. Scale bar, 50 μm. (**D,E**) NC, MCF10A-shDLG5, MCF7-shDLG5 and MDA-MB-231-oxDLG5 cells were stained with PI and analyzed by flow cytometry (BD FACSCanto II) at 488 nm. (**F,G**) NC, MCF10A-shDLG5, MCF7-shDLG5 and MDA-MB-231-oxDLG5 cells were stained with BrdU-PerCP and analyzed by flow cytometry (FACSCanto II) at 488 nm. (**H**) The expression of cell proliferation-associated proteins, including cyclin D1, p21, p27 and p53, and the phosphorylation of ERK1/2 in NC, MCF10A-shDLG5, MCF7-shDLG5 and MDA-MB-231-oxDLG5 cells were analyzed by western blot.

Classical DLG proteins and ZO subfamily members such as DLG1^22^, DLG3^23^, ZO1, ZO2 and ZO3^24^,^25^ have been reported to be associated with cell proliferation. DLG5 possesses a structure similar to classical DLG proteins and ZO subfamily members^26^,^27^, suggesting that DLG5 may also play a role in cell proliferation. To prove our hypothesis, we constructed DLG5-knockdown MCF10A and MCF7 cell lines (MCF10A-shDLG5 and MCF7-shDLG5), a DLG5-overexpressing MDA-MB-231 cell line (MDA-MB-231-oxDLG5) and respective negative control (NC) cell lines by lentivirus-mediated shRNA interference (Fig. 2A). Analyses using a Live Cell Imaging System and by cell counting showed that DLG5-knockdown cells had a higher growth rate than NC cells. After 24 hrs, there were nearly twice as many MCF7-shDLG5 cells as NC cells. After 48 hrs, MCF7-shDLG5 cells were at almost 100% confluence, but NC cells were at approximately 50% confluence. The opposite results were observed in MDA-MB-231-oxDLG5 cells (Fig. 2B,C; supplementary movies 1, 2).

A subsequent study in MCF10A-shDLG5 and MCF7-shDLG5 cells revealed an increased percentage of cells in S phase and G2/M phase and a decreased percentage in G1 phase; conversely, DLG5 overexpression in MDA-MB-231 cells inhibited cell cycle progression (Fig. 2D,E). MCF10A-shDLG5 and MCF7-shDLG5 cells showed a higher rate of BrdU incorporation than NC cells. In contrast, percentage of BrdU positive cells in MDA-MB-231-oxDLG5 cells was lower than that of NC cells (Fig. 2F,G). The results indicate that loss of DLG5 may promote cell proliferation by increasing DNA synthesis during S phase.

We also examined the expression of cell proliferation-associated proteins following a change in DLG5 expression. Loss of DLG5 expression induced the up-regulation of cyclin D1 expression and the significant down-regulation of p21, p27 and p53 expression in MCF10A-shDLG5 and MCF7-shDLG5 cells, while overexpressing DLG5 resulted in reduced cyclin D1 expression and increased p21, p27 and p53 expression in MDA-MB-231-oxDLG5 cells (Fig. 2H).

Extracellular signal-regulated kinase (ERK) 1/2 participates in the Ras-Raf-MEK-ERK signal transduction cascade and is involved in regulating cancer development and progression, cell adhesion, cell proliferation, and cell migration^28^. We found that MCF10A-shDLG5 and MCF7-shDLG5 cells showed higher ERK1/2 phosphorylation levels than their respective NC cells. In contrast, up-regulating DLG5 expression in MDA-MB-231 cells reduced ERK1/2 phosphorylation levels (Fig. 2G). The results imply that DLG5 potentially accelerates cell proliferation by up-regulating cyclin D1; down-regulating p21, p27 and p53; and enhancing Ras-Raf-MEK-ERK signaling.

### DLG5 knockdown disrupts breast cell acinar polarity

**Figure 3:**
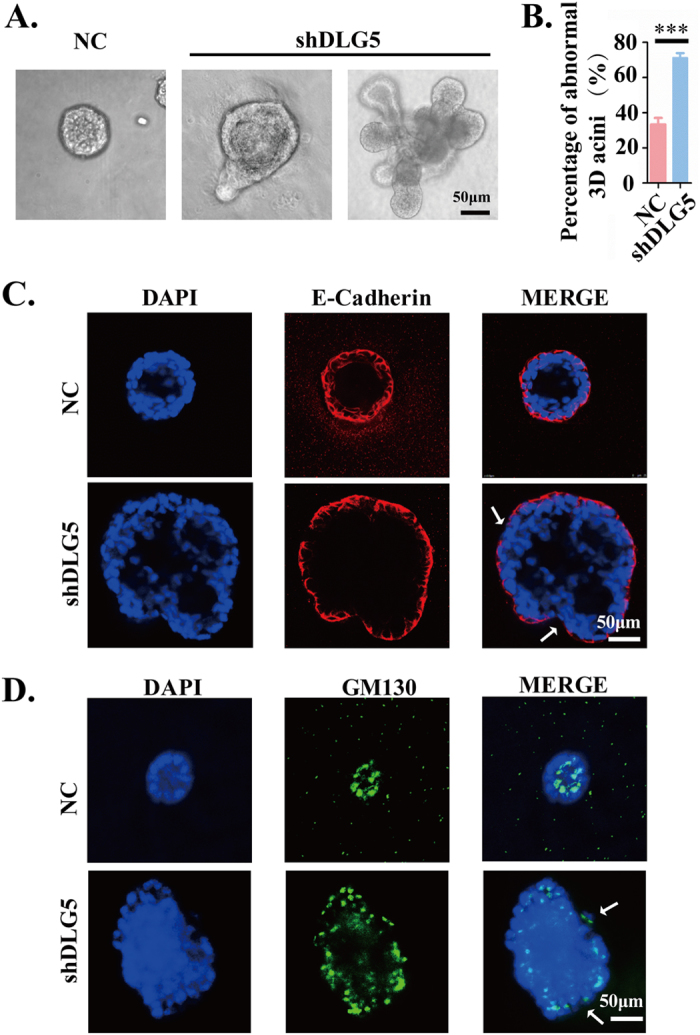
Loss of DLG5 disrupts acinar morphogenesis in 3D culture. (**A,B**) MCF10A cells were cultured in matrigel and imaged after 16 days (scale bar, 50 μm). (**C,D**) Immunofluorescence analysis of E-cadherin and GM130 in 3D acini. Scale bar, 50 μm.

The morphogenesis of NC and MCF10A-DLG5 cells in 3D culture was studied to determine the exact role of DLG5 in the maintenance of cell polarity. Under 3D culture conditions, MCF10A cells proliferate and organize into spheroids, commonly called “acini”^12^. These acini have a central lumen and are lined by layers of polarized epithelial cells. We cultured MCF10A-shDLG5 cells and NC cells in 3D culture for 16 days. DLG5-knockdown cells failed to form acini or formed acini with disordered morphogenesis compared with NC cells (Fig. 3A,B). E-cadherin exhibited a continuous ring-like distribution on the outer layer of the acini formed by MCF10A-NC cells. DLG5 knockdown induced a partial loss of E-cadherin expression and discontinuous E-cadherin distribution on the outer layer of the acini (Fig. 3C). GM130 is regarded as a biomarker of the orientation of the Golgi apparatus toward the lumen in 3D morphogenesis studies; therefore, GM130 mislocalization represents a change in cell polarity^29^. In response to loss of DLG5 expression, GM130 was translocated to the outside surface of the acini (Fig. 3D). These results indicate that DLG5 plays an important role in the maintenance of cell polarity of breast acini.

### Loss of DLG5 induces EMT and promotes cell migration and invasion

**Figure 4:**
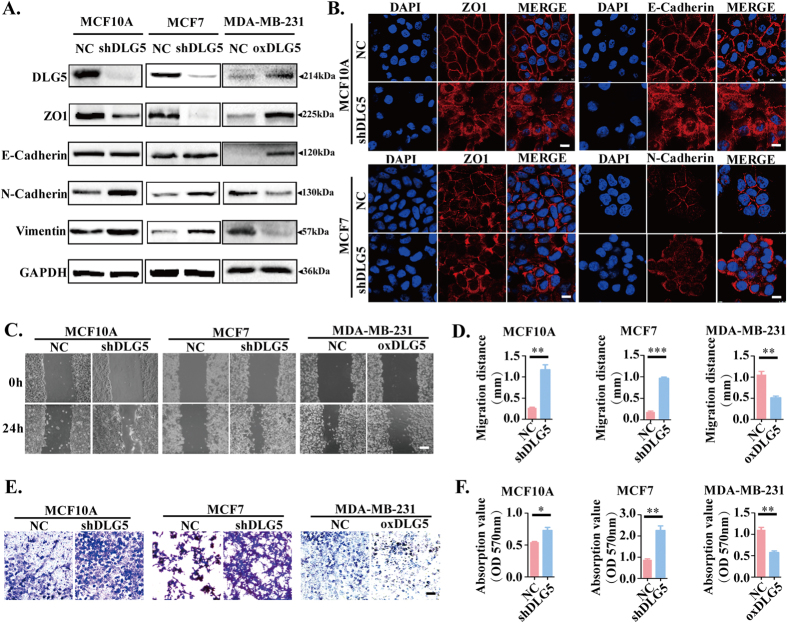
Knockdown of DLG5 induces EMT and cell migration and invasion. (**A**) Western blot analysis of EMT makers. (**B**) Immunofluorescence analysis of ZO1 and E-cadherin localization in NC and MCF10A-shDLG5 cells and of ZO1 and N-cadherin localization in NC and MCF7-shDLG5 cells. Scale bar, 25 μm. Monolayer wound healing assay (**C,D**: scale bar, 100 μm) and transwell assay (**E,F**: scale bar, 50 μm) of NC, MCF10A-shDLG5, MCF7-shDLG5 and MDA-MB-231-oxDLG5 cells.

Failure to maintain cell polarity is always accompanied by EMT^30^,^31^. To investigate the role of DLG5 in EMT in breast cancer cells, we compared the expression of EMT makers in MCF10A-shDLG5, MCF7-shDLG5 and NC cells. We found that DLG5 knockdown altered the expression of certain EMT markers; the expression of the mesenchymal markers N-cadherin and vimentin was increased, that of the epithelial marker ZO1 was decreased, and that of the epithelial marker E-cadherin did not change. In MDA-MB-231-oxDLG5 cells, there was a notable increase in ZO1 and E-cadherin expression and a decrease in N-cadherin and vimentin expression (Fig. 4A). Additionally, ZO1, E-cadherin and N-cadherin localized at both cell-cell junctions and in the cytoplasm after DLG5 knockdown (Fig. 4B). The results indicate that loss of DLG5 might partially induce EMT.

In our experiments, we noticed that MCF7-shDLG5 cells exhibited greater mobility than NC cells (supplementary movies). To identify the role of DLG5 in cell mobility, we performed monolayer wound healing and transwell assays with MCF10A-shDLG5 and MCF7-shDLG5 cells. DLG5 knockdown led to increased cell migration without an effect on cell proliferation (Fig. 4C,D). Loss of DLG5 expression also increased the invasive ability of MCF10A and MCF7 cells compared with NC cells (Fig. 4E,F). Overexpressing DLG5 in MDA-MB-231 cells decreased migration (Fig. 4C,D) and invasion (Fig. 4E,F). As shown in Fig. 1, DLG5 was more highly expressed in the luminal subtype cell lines (MCF7, T47D and BT474) than in the basal-like metastatic cell lines (MDA-MB-468, MDA-MB-231 and MDA-MB-453). Taken together, loss of DLG5 expression induced partial EMT and enhanced cell migration and invasion; DLG5 might also be involved in regulating the metastatic progression of breast cancer.

### Loss of DLG5 inactivates the Hippo signaling pathway

**Figure 5:**
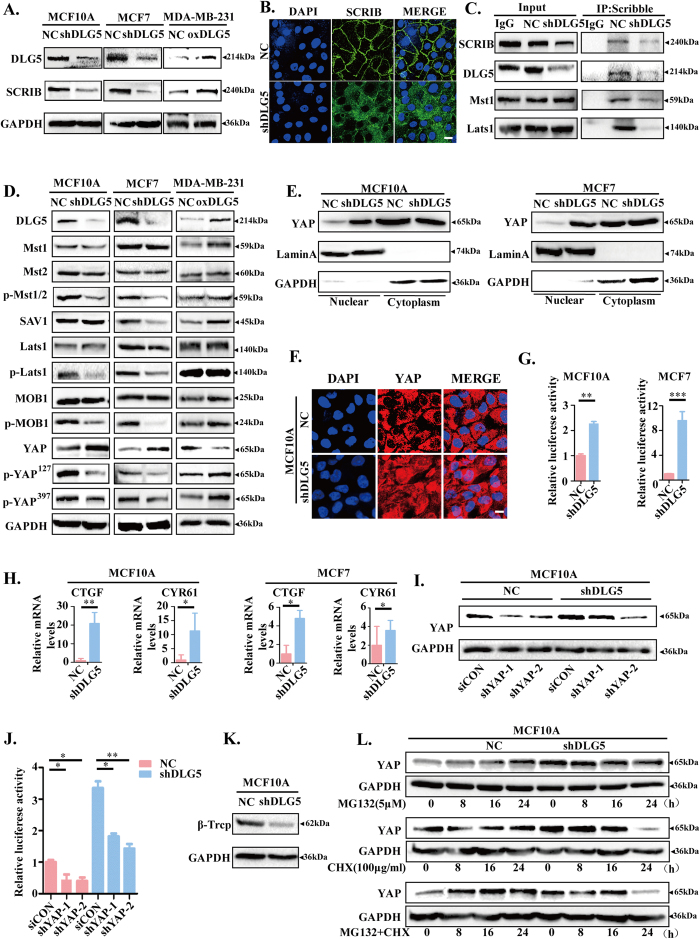
Loss of DLG5 inhibits the Hippo signaling pathway. (**A**) Western blot analysis of SCRIB in NC, MCF10A-shDLG5, MCF7-shDLG5 and MDA-MB-231-oxDLG5 cells. (**B**) Immunofluorescence analysis of SCRIB localization in NC and MCF10A-shDLG5 cells. Scale bar, 25 μm. (**C**) Co-IP assay and western blot analysis of the interaction of DLG5, MST1, and Lats1 with SCRIB in NC and MCF10A-shDLG5 cells. (**D**) Western blot analysis of Hippo signaling pathway components in NC, MCF10A-shDLG5, MCF7-shDLG5, and MDA-MB-231-oxDLG5 cells. (**E,F**) Western blot analysis and immunofluorescence analysis of the nuclear and cytoplasmic distribution of YAP in NC and MCF10A-shDLG5 cells. Scale bar, 25 μm. (**G**) TEAD activity was analyzed by luciferase assay. (**H**) The relative mRNA levels of CTGF and CYR61 were analyzed by RT-PCR. I. Western blot analysis of YAP expression following siRNA-mediated YAP knockdown. (**J**) TEAD activity was analyzed by luciferase assay following siRNA-mediated YAP knockdown. (**K**) Western blot analysis of β-Trcp expression in NC and MCF10A-shDLG5 cells. (**L**) NC and MCF10A-shDLG5 cells were treated with MG132 (5 μM) and/or CHX (100 μg/ml) for 8, 16, and 24 hrs. Extracts were analyzed by western blot.

Scribble is required for Hippo signaling pathway activity in both *Drosophila* and mammalian cells^32^. Knockdown of Scribble leads to increases in YAP nuclear localization and TEAD transcription owing to the absence of the interactions of Mst1/2 and Lats1/2 with Scribble, which ultimately inactivates the Hippo signaling pathway^33^,^34^. ZO2 is also a member of the MAGUK family and is crucial in cell-cell interactions; activation of the PDZ domain in ZO2 can facilitate the nuclear localization of YAP^35^. DLG5 has a similar structure as ZO2 and contains 4 PDZ domains. In our study, Scribble showed significantly decreased expression in MCF10A-shDLG5 and MCF7-shDLG5 cells and increased expression in MDA-MB-231-oxDLG5 cells (Fig. 5A). Additionally, we observed increased mislocalization of Scribble in MCF10A-shDLG5 cells (Fig. 5B). Interestingly, co-IP assays showed that DLG5 interacted with Scribble (Fig. 5C). Loss of DLG5 expression impaired the interactions of Mst1/2 and Lats1/2 with Scribble. The results suggest that DLG5 might be an upstream regulator of the Hippo signaling pathway.

To ascertain whether DLG5 has an effect on YAP expression and localization, a downstream effector of the Hippo signaling pathway, we examined the expression of the major members of the Hippo signaling pathway in MCF10A-shDLG5 and MCF7-shDLG5 cells. We found that loss of DLG5 expression decreased the levels of p-MST1/2, SAV1, p-MOB1, p-LAST1, p-YAP^S127^ and p-YAP^S397^ (Fig. 5D). YAP phosphorylation on Ser127 was also decreased, which resulted in reduced YAP-14-3-3 binding and YAP cytoplasmic retention^36^. A subsequent study revealed apparently increased nuclear YAP and TEAD transcription in MCF10A-shDLG5 and MCF7-shDLG5 cells compared with NC cells (Fig. 5E,F,G). Furthermore, loss of DLG5 expression increased the expression of CTGF and CYR6, two genes downstream of TEAD (Fig. 5H). As predicted, knockdown of YAP in MCF10A-shDLG5 cells decreased TEAD transcriptional activity (Fig. 5I,J).

Interestingly, loss of DLG5 not only increased YAP expression but also decreased p-YAP^S397^ levels (Fig. 5D). A recent report showed that β-Trcp recognizes YAP phosphorylation on Ser397 and triggers YAP degradation^37^. We detected a significant decrease in β-Trcp expression in MCF10A-shDLG5 cells (Fig. 5K). In contrast, in NC cells, loss of DLG5 promoted YAP stabilization when protein synthesis was not blocked with cycloheximide (CHX). Additionally, YAP accumulated in NC cells, but not in shDLG5 cells, indicating that loss of DLG5 expression might inhibit YAP degradation (Fig. 5L). Taken together, these data demonstrate that loss of DLG5 expression inhibits the Hippo signaling pathway and increases the expression and nuclear localization of YAP.

**Figure 6:**
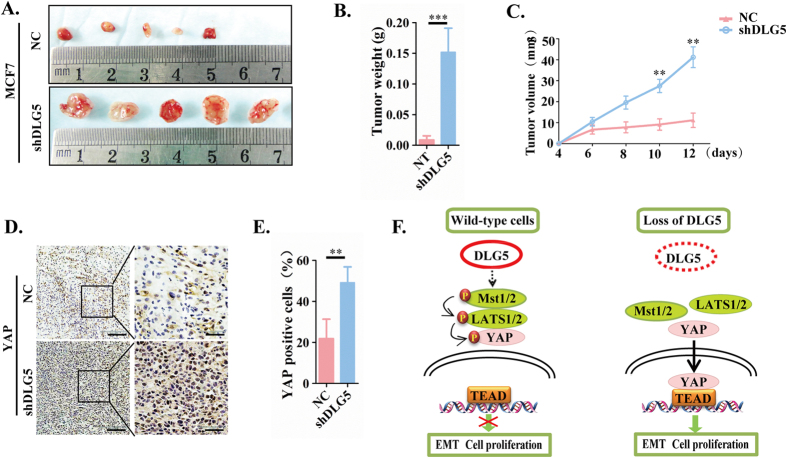
Loss of DLG5 induces tumor proliferation *in vivo*. (**A**) NC or MCF 7-shDLG5 cells (5 × 10^6^) were subcutaneously injected into nude mice. Tumors were excised, and images were taken after 12 days. Tumor weight (**B**) was measured after 12 days, and tumor volume (**C**) was recorded every two days after tumor inoculation. (**D**). YAP and SCRIB localization was analyzed by immunostaining. Low-power scale bar, 150 μm; high-power scale bar, 50 μm. (**F**) Schematic of the role of DLG5 in breast cancer progression to malignancy.

*In vivo* studies showed similar results. MCF7-NC or MCF7-shDLG5 cells were subcutaneously injected into nude mice. Knockdown of DLG5 increased tumor weight and volume (Fig. 6A,B,C) and elicited a remarkable increase in YAP and a decrease in Scribble (Fig. 6D,E). Both the *in vitro* and *in vivo* results showed that knockdown of DLG5 inactivated the Hippo signaling pathway by decreasing Scribble expression and leading to its mislocalization in the cytoplasm, consequently inducing EMT, loss of cell polarity, cell migration and cell proliferation (Fig. 6F).

## Discussion

We are the first to report that loss of DLG5 increased YAP nuclear localization, inhibited YAP degradation, down-regulated Scribble expression, and mislocalized Scribble from the membrane to the cytoplasm; these findings provide insight into the mechanism underlying breast cancer progression. Specifically, loss of DLG5 down-regulated and mislocalized Scribble, inhibited the Hippo signaling pathway by decreasing the interaction of Scribble with Mst1 and Lats1, and increased YAP nuclear localization and TEAD transcription, thereby promoting the proliferation of breast epithelial cells and breast cancer cells, inducing EMT, disrupting the maintenance of cell polarity, and increasing cell migration and invasion.

DLG5 was highly expressed in normal breast tissues/cells and low-grade breast cancer tissues/cells, but its expression was reduced or lost in high-grade breast cancer tissues/cells. Obviously, DLG5 is closely related to breast cancer development. In addition, we showed that DLG5 expression is positively associated with ER/PR status, an important indicator for breast cancer therapy and prognosis. Previous studies have reported that DLG5 is a primary target of progesterone and shows high expression in luminal breast cancer^8^,^38^. These results suggest that DLG5 may be correlated with cancer therapy and prognosis.

In 1998, Nakamura *et al*. reported that DLG5 may regulate cell proliferation^39^, but these researches did not ascertain the detailed role of DLG5 in this process. We found that DLG5 expression positively correlated with p53 expression and was negatively associated with Ki67 expression. Both p53 and Ki67 are notable markers of cell proliferation^17^. Interestingly, we also observed that DLG5-knockdown cells grew faster than NC cells. Our results verified the conclusion by Nakamura *et al*. that DLG5 regulates cell proliferation. p53 has been reported to play a paramount role in inducing G1 and G2/M arrest. Loss of p53 expression causes cells to avoid cell cycle progression checkpoints and induces cell proliferation and tumorigenesis^18^. p21 and p27 are important inhibitors of G1 progression and hallmarks of cell overgrowth^40^. Overexpression of cyclin D1 enables cells to pass the G1/S checkpoint and thus increases the percentage of cells in S phase^41^. According to previous reports, overexpression of cyclin D1 and loss of p53, p21 and p27 expression induce cell overgrowth and are significant markers of malignant cancer^18^,^42^,^43^. Our study generated similar results; DLG5 knockdown reduced p53, p21 and p27 expression and increased cyclin D1 expression in MCF10A-shDLG5 and MCF7-shDLG5 cells. Taken together, loss of DLG5 promotes tumor malignancy by regulating cell overgrowth.

DLG5 is involved in regulating the maintenance of epithelial polarity, EMT, and cell migration and invasion^8^. We found that DLG5 co-localized with E-cadherin at cell-cell junctions in the MCF10A breast epithelial cell line. Knockdown of DLG5 disrupted breast cell acinar polarity by inducing the mislocalization of E-cadherin and GM130. Additionally, loss of DLG5 expression induced EMT by altering the localization and expression of EMT markers, specifically by decreasing ZO1 expression and increasing N-cadherin and vimentin expression. However, E-cadherin expression did not change in response to changes in DLG5 expression. Our results suggest that loss of DLG5 might disrupt the maintenance of cell polarity, accompanied by partial EMT.

The Hippo signaling pathway is an evolutionarily conserved regulator of cell proliferation, cell polarity maintenance and tumor suppression. Alterations in this pathway are increasingly recognized to be associated with cancer development^2^,^5^,^44^. The mammalian core kinase components comprise MST1/2, SAV1, LATS1/2 and MOB1^34^,^44^. YAP is a downstream effector of the Hippo pathway, and nuclear YAP is involved in regulating cell proliferation, EMT, and cell polarity maintenance^45^. Phosphorylation of YAP on Serine 127 is crucial for promoting its association with 14-3-3 protein and its subsequent cytoplasmic sequestration and inactivation^3^. YAP phosphorylation on Ser397 is recognized by β-Trcp, an F-box protein that participates in the ubiquitination and degradation of YAP^37^. Previous research showed that DLG5 expression is down-regulated in YAP-overexpressing cells^8^. Recent studies revealed that many cell polarity proteins, such as LKB1 and ZO2, play an important role in regulating the Hippo signaling pathway^35^,^46^. Thus, we were curious whether DLG5 expression has an effect on the Hippo signaling pathway. We first found that loss of DLG5 expression decreased the levels of p-MST1/2, SAV1, p-MOB1, and p-LAST1, resulting in inactivation of the Hippo signaling pathway. Loss of DLG5 expression decreased p-YAP^S127^ and p-YAPS^397^ levels, thereby inhibiting YAP degradation and increasing YAP nuclear localization.

A recent report showed that Scribble acts downstream of LKB1 to regulate the Hippo signaling pathway^46^. Scribble expression and localization to the plasma membrane are key factors in the activation of the Hippo signaling pathway. Impaired interactions of Mst1 and Lats1 with Scribble inhibit the Hippo signaling pathway and induce the nuclear localization of YAP. We found that loss of DLG5 expression inhibited Scribble expression and induced its mislocalization to the cytoplasm. The interactions of Mst1 and Lats1 with Scribble were decreased in MCF10A-shDLG5 and MCF7-shDLG5 cells. Taken together, loss of DLG5 expression promoted breast cancer malignancy by inactivating the Hippo pathway and increasing nuclear YAP.

In conclusion, loss of DLG5 expression (1) inhibits the Hippo pathway by inducing the mislocalization of Scribble and decreasing its expression; (2) increases YAP nuclear localization; and (3) suppresses p21, p27 and p53 expression. In general, loss of DLG5 expression promotes cell proliferation, EMT, maintenance of cell polarity, and cell migration and invasion by inactivating the Hippo signaling pathway. We expect that our findings may offer a potential therapeutic target for breast cancer.

### Ethical approval and consent to participate

All patients signed informed consent forms, and the study was approved by the Ethics Review Committee of the First Affiliated Hospital of Xi’an Jiaotong University College of Medicine. All animal experiments were conducted according to the guidelines of the Institutional Animal Care and Use Committee of the First Affiliated Hospital of Xi’an Jiaotong University.

## Additional Information

**How to cite this article:** Liu, J. *et al*. Loss of DLG5 promotes breast cancer malignancy by inhibiting the Hippo signaling pathway. *Sci. Rep.* **7**, 42125; doi: 10.1038/srep42125 (2017).

**Publisher's note:** Springer Nature remains neutral with regard to jurisdictional claims in published maps and institutional affiliations.

## Supplementary Material

Supplementary Information

Supplementary Movie 1

Supplementary Movie 2
