# Supplementary material for: Loss of DLG5 promotes breast cancer malignancy by inhibiting the Hippo signaling pathway
Source: Sci Rep. 2017 Feb 7;7:42125. doi: 10.1038/srep42125 (PMC5294562; doi:10.1038/srep42125)
Supplement: Supplementary Information [file srep42125-s1.doc]

Loss of DLG5 promotes breast cancer malignancy by inhibiting the Hippo signaling pathway

**Jie Liu^1, 2, #^, Juan Li^1, 2, #^, Pingping Li^1, 2^, Yaochun Wang^1, 2^, Zheyong Liang^1, 2^, Yina Jiang^3^, Jing Li^1, 2^, Chen Feng^4^, Ruiqi Wang^1, 2^, He Chen^1, 2^,** **Can Zhou^5^, Jianmin Zhang^6^, Jin Yang ^7^ & Peijun Liu^1, 2, *^**

^1^ Center for Translational Medicine, the First Affiliated Hospital of Xi’an Jiaotong University, Xi’an, Shaanxi, China

^2^ Key Laboratory for Tumor Precision Medicine of Shaanxi Provincer, the First Affiliated Hospital of Xi’an Jiaotong University, Xi’an, Shaanxi, China

^3^ Department of Pathology, the First Affiliated Hospital of Xi’an Jiaotong University, Xi’an, Shaanxi, China

^4^ Department of Oncology, the Shaanxi Provincial Corps’ Hospital, Xi’an, Shaanxi, China

^5^ Department of Breast Surgery, The First Affiliated Hospital of Xian Jiaotong University, Xi’an, Shaanxi 710061, China

^6^ Department of Cancer Genetics, Roswell Park Cancer Institute, Buffalo, New York, USA

^7^ Department of Medical Oncology, the First Affiliated Hospital of Xi’an Jiaotong University, Xi’an, Shaanxi, China

^#^Contributed equally

^*^Corresponding author, Peijun Liu, Center for Translational Medicine, the First Affiliated Hospital of Xi’an Jiaotong University, 277 West Yanta Road, Xi’an, Shaanxi 710061, China. Tel: +86 189 9123 2306; Fax: +86 029 85324628; E-mail: liupeijun@xjtu.edu.cn

**Supplementary Information**

**sTable. 1.** Clinical profile of patients


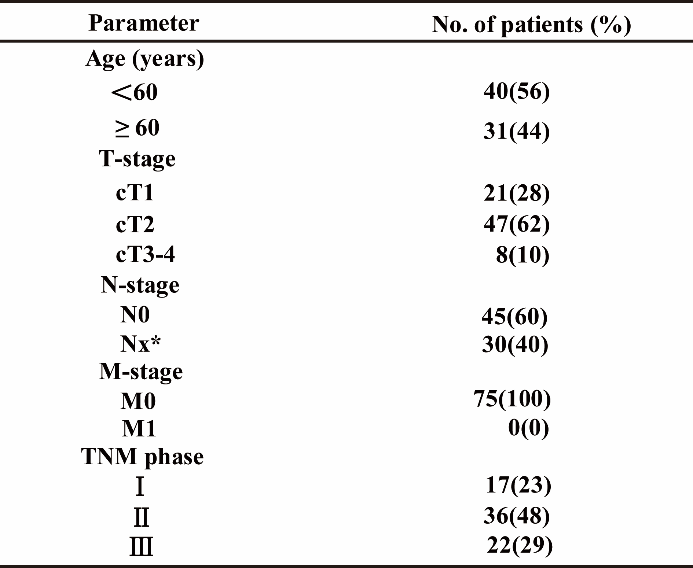


**sTable. 2.** Association of DLG5 immunoreactivity with clinical stages and clinic pathologic features of human breast cancer


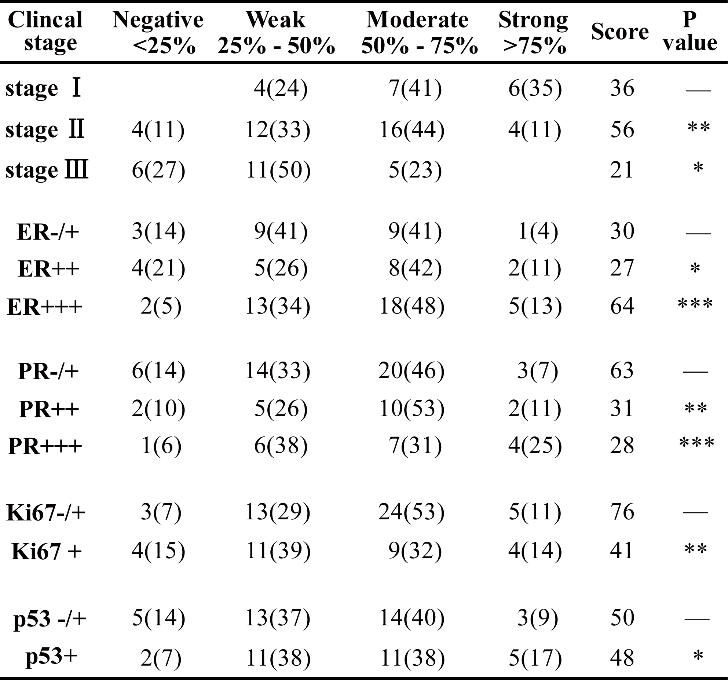


**Supplementary Movie 1**: MCF7-NC cells were photographed by Live Cell Imaging System to examine their growth status.

**Supplementary Movie 2**: MCF7-shDLG5 cells were photographed by Live Cell Imaging System to examine their growth status.
